# Agranulocytosis under biotherapy in rheumatoid arthritis: three cases hypothesis of parvovirus B19 involvement in agranulocytosis observed under tocilizumab and rituximab for the treatment of rheumatoid arthritis

**DOI:** 10.1007/s10067-016-3379-6

**Published:** 2016-08-19

**Authors:** C. Giraud, Z. Tatar, M. Soubrier

**Affiliations:** 1 Department of Rheumatology, Hôpital Gabriel Montpied, 58 Montalembert street, 63000 Clermont-Ferrand, France; 2 Department of Rheumatology, Hôpital Gabriel Montpied, 58 rue Montalembert street, 63000 Clermont-Ferrand, France

**Keywords:** Agranulocytosis, Drug-related side effects and adverse reactions, Human, Parvovirus B19, Rheumatoid arthritis

## Abstract

Leukopenia is a considerably common complication of tocilizumab [TCZ] and rituximab [RTX] therapy. RTX-induced leukopenia typically exhibits delayed onset. While agranulocytosis has been reported linked to RTX treatment of lymphoma, this complication rarely occurs in rheumatoid arthritis (RA) treatment and, to our knowledge, has never been reported in association with TCZ therapy. We herein report four agranulocytosis cases in three patients, with the first two cases suspected to be secondary to human parvovirus B19 (PVB19) infection. Agranulocytosis manifested in the first patient 2 months following a third RTX course. Bone marrow (BM) polymerase chain reaction (PCR) was positive for PVB19. The patient relapsed after three TCZ courses, with her PCR again positive for PVB19. Both episodes resolved under granulocyte-macrophage colony-stimulating factor (GM-CSF). In the second patient, agranulocytosis manifested after the 74th TCZ course. Bone marrow PCR was positive for PVB19, and the evolution was favorable under intravenous immunoglobulin administration. The third case was a 53-year-old female patient with seropositive RA who presented agranulocytosis after the first infusion of her fourth RTX course. Unfortunately, no PCR PVB19 was made on myelogram. Evolution was favorable after 5 days of GM-CSF. PVB19 infection should be investigated in patients suffering from agranulocytosis manifesting during biotherapy. In cases manifesting from the 15th day of RTX treatment onwards, hemogram must be conducted before readministering the infusion.

## Introduction

Prognosis of rheumatoid arthritis (RA) has been radically changed since the introduction of biologic disease-modifying antirheumatic drug. Among the adverse events of these treatment, leukopenia is common under tocilizumab (TCZ) and rituximab (RTX) [1–4]. However, agranulocytosis defined by neutrophils count <0.5 G/L on hemogram remains rare. Agranulocytosis has been already reported manifesting during RTX therapy for lymphoma (as late-onset neutropenia, LON); yet to our knowledge, this complication is less common in RA treatment and has never been reported during TCZ therapy[4–7]. The incidence and etiologies of LON are largely discussed in literature. The overall incidence, calculated from series published in hematology, was estimated at 3–27% [6]. In RA, the incidence was estimated at 1.3% of all treated patients [7], caused by blocked medullary granulocytic maturation occurring 3 to 4 weeks after RTX infusion. The potential causes found are drug toxicity, antigranulocyte antibody production, neutrophil apoptosis by the large granular lymphocyte population, polymorphisms in the IgG receptor FC γ RIIIA, SDF 1 synthesis during lymphocyte B recovery, as well as viral infection [5–9]. Among the viral pathogens, human parvovirus B19 could be a causative agent (PVB19). PVB19 infection is known to induce transient aplasia. It can also cause leukopenia and agranulocytosis in both healthy and immunocompromised individuals[10–12]. We herein report four agranulocytosis episodes in three RA patients undergoing TCZ or RTX therapy. PVB19 infection was detected in two of our observed cases and could have accounted for the agranulocytosis observed under biotherapy.

## Clinical cases

### Case 1

This 32-year-old female had a history of cerebellar ataxia and seronegative RA that started in 2004. Following the failure of three antitumor necrosis factor alpha (anti-TNFα) agents, namely adalimumab, infliximab, and etanercept combined with methotrexate (MTX), RTX was administered in November 2006. Treatment was recommenced in November 2008 and November 2009. MTX was discontinued in December 2010 then RTX administered in February 2011. Two months later, a full blood count (FBC) performed due to fever revealed isolated agranulocytosis (0.03 G/L) (hemoglobin (Hb): 12.2 g/dL, platelets: 339 G/L) with severe inflammatory syndrome (C-reactive protein (CRP): 110 mg/L). The infection work-up was negative: urine cultures revealed no growth; pneumococcal and legionella urinary antigen tests were negative; tests for mycoplasma pneumoniae, chlamydia pneumoniae, HIV, hepatitis B virus (HBV), hepatitis C virus (HCV), and cytomegalovirus (CMV) were negative; Epstein-Barr virus (EBV): IgG positive and IgM negative; and parvovirus: IgG positive and IgM negative. The patient’s morphological examinations were normal. Myelogram revealed precursor block at the promyelocytic stage. Polymerase chain reaction (PCR) was positive for PVB19 in the bone marrow (BM) and negative in the blood. Granulocyte-macrophage colony-stimulating factor (GM-CSF) treatment was administered for 48 hours. Leukocytes normalized by day 4. In January 2012, the RA flared up again, and TCZ therapy was initiated. FBC performed prior to the fourth treatment course revealed agranulocytosis (neutrophils: 0 G/L). The clinical examination was normal. Myelogram demonstrated precursor block in the promyelocytic stage, and the PCR once again detected PVB19. The patient’s infection work-up was negative, and she improved within 72 hours of receiving GM-CSF.

### Case 2

This 70-year-old female had a history of recurring pulmonary embolism and suffered from a seronegative RA, diagnosed in 1969. Firstly, she was treated with aurothiopropanol then sulfasalazine and MTX with hydroxychloroquine. She was also given corticosteroids. In 2001, her RA flared up. Anti-TNFa treatment was initiated, replaced in 2007 by abatacept until 2009. Due to a new RA episode, TCZ was started (8 mg/kg) in association with MTX in 2009. There were no infusion complications. In 2015, 1 month after the last infusion (75^th^), neutropenia occurred, with a neutrophil level of 0.1 G/L declining to 0.08 G/L in 1 week. The patient’s clinical examination was normal. Other biological tests disclosed aregenerative anemia (Hb: 9.7 g/dL, reticulocytes: 85.2 G/L), with lymphopenia (0.360 G/L), while platelet count was normal (247 G/L). Serological tests revealed no recent viral infection (CMV: IgG+, IgM−; EBV: IgG+, IgM−; PVB19: IgG+, IgM−). PCR was positive for PVB19 in the bone marrow: 65 cop/mL (>42) yet negative in the blood. No vitamin B12 or folate deficiencies were identified, with normal liver and kidney analyses. There was no inflammation, and blood cultures were negative. Myelogram revealed normal abundance of three lineages with a blockade of neutrophil lineage at the promyelocyte/myelocyte stage, which indicated agranulocytosis. All treatments (MTX and TCZ) were stopped. Intravenous immunoglobulins (IgIV) were infused at 1 mg/kg for 2 days. There were no complications. Three days post-infusion, the polynuclear neutrophil (PNN) level increased and remained constant at 1.7 G/L. TCZ was reinitiated 7 days after agranulocytosis at half the original dosage in association with MTX, resulting in no further hematological complications.

### Case 3

This 53-year-old female displayed no relevant medical history except for thyroidectomy due to goiter and seropositive RA diagnosed in 2005. She was initially treated with MTX then an MTX-adalimumab combination in 2005, followed by TCZ and etanercept in 2011, each discontinued in turn due to insufficient efficacy. RTX was introduced in March 2012 and again in November 2012 and April 2013. Hydroxychloroquine was added due to persistent RA flare-up in February 2013. In November 2013, the patient received 1 g of RTX. Her FBC, performed prior to the second infusion, revealed isolated neutropenia (0.089 G/L). The patient was apyretic despite left maxillary sinusitis, which was resolving. Myelogram revealed neutrophilic granulocytic hypoplasia with blocked promyelocytic maturation, along with an absence of mature elements, suggesting agranulocytosis. Viral serology (HIV, HBV, HCV, EBV, and PVB19) was negative in the blood. No parvovirus PCR was performed in the bone marrow. All treatments were discontinued. The patient’s condition evolved favorably within 5 days of initiating GM-CSF.

## Discussion

We herein report four agranulocytosis episodes manifesting in three patients under biotherapy, with two of the episodes being possibly secondary to PVB19 infection. The first patient’s PCR was positive for PVB19 when she developed agranulocytosis while receiving RTX then TCZ. In the second episode, the PCR was also positive when agranulocytosis manifested under TCZ.

Neutropenia is a common complication of TCZ and RTX treatment in RA patients. The *LITHE, STREAM*, and *AMBITION* studies observed 17 (4.3 %), nine (6.2 %), and nine (3.1 %) cases of stage 3 neutropenia (PNN 0.5–1 G/L), respectively, in TCZ-treated patients receiving 8 mg/kg [1–3]. Temporarily blocked demargination of the polynuclear neutrophils, usually mediated by IL6, appeared responsible, with no bone marrow abnormalities detected [13]. Agranulocytosis has never been reported associated with TCZ use in RA.

Under RTX, most cases of neutropenia secondary to infusion are described as late-onset neutropenia (LON), notably in hematological series [7]. Marotte et al. are the first authors who reported agranulocytosis in an RA patient manifesting 8 weeks after the first RTX infusion. They detected a blocked granulocytic maturation on myelogram, with favorable evolution achieved on initiating GM-CSF. The underlying neutropenia mechanism remained unclear, with low residual RTX concentrations and absence of antigranulocyte antibodies. Virus was thus suspected, notably PVB19 [5]. The possibility of RTX, agranulocytosis, and PVB19 association has already previously been raised in one neutropenia case in a patient treated for primary biliary cirrhosis [14].

Parvovirus B19 uses the human erythroid progenitor for natural host cells[11]. Pure red-cell aplasia is the most common feature, while some other hematological complications may also occur. Although erythroid progenitors appear specific permissive cells for PVB19 replication, neutropenia with agranulocytosis and thrombocytopenia or pancytopenia has also been reported in the literature [11, 12, 17]. A primo-infection as well as virus reactivation can induce neutropenia. In immunocompromised patients, reactivation of PV B19 detected by PCR can occur at low levels of parvovirus replication owing to absent antiviral immunity [16]. Several publications have testified to an association between neutropenia, agranulocytosis, and PVB19 in both healthy and immunocompromised patients. McClain K. et al. and Istomin V. et al. reported 15 PVB19-positive PCRs in 19 chronic neutropenia children and five agranulocytosis cases in 23 patients with acute PVB19 infection, respectively [15, 17]. Two pure agranulocytosis cases, secondary to PVB19 infection, were also reported by Pont and Herzog-Tzarfati [18, 19]. In immunocompromised cases, comparable to our three cases, Barlow et al. reported that the majority of the 26 documented cases of PVB19-related neutropenia occurred in hemopathic or immunocompromised patients [20]. In a case of late-onset neutropenia (LON) under RTX, Hartman et al. described one patient treated for lymphoma presenting negative blood tests, with only her blood and bone marrow PCRs testing positive, and no other abnormalities indicating PV infection [21]. Her evolution was favorable under IgIV administration. In addition, Christopeit et al. described PV infection being responsible for a LON case, with only the bone marrow PCR positive for PV [22]. This context is comparable to our first case, where the PV infection could have been responsible for the agranulocytosis under RTX, as well as the relapse under TCZ, with the latter inducing immunodepression via antilymphocyte B activity, thus explaining the agranulocytosis [23]. Unfortunately, no test for PV B19 on bone marrow was performed to our third case. We can only present the occurrence of agranulocytosis 2 weeks after RTX infusion but can not confirm parvovirus responsibility.

The association between agranulocytosis and PVB19 remains complex, and the viral replication’s exact role in neutropenia pathogenesis is still unclear [24–26]. PVB19 may either inhibit myeloid cell development or exhibit a direct cytotoxic effect [25]. Induction of antigranulocyte antibodies was also considered. In a study on 240 patients with autoimmune neutropenia, 36 exhibited seroconversion and/or positive DNA in the blood [27]. Of these 36, 24 were retested after neutropenia remission and all remained negative for parvovirus [27]. In our cases, this hypothesis could not explain the neutropenia. We searched for antigranulocyte antibodies, but all were negative, whereas the PVB19 serology and DNA were positive in either the blood or bone marrow.

Lastly, to treat infection or reactivation, at least temporarily discontinuing immunosuppressive therapy is advised. Granulocyte-stimulating factors should primarily be used in cases of general neutropenia that last a few days. In such resistant or severe cases, intravenous immunoglobulin can provide antibodies against PVB19 and proves highly effective. Such a regimen should be administered at either 0.4–0.5 g/kg per day during 5 to 10 days or 1 g/kg for 2 days [10, 18]. The PCR should subsequently become negative for PVB19. Neutropenia relapse may require new immunoglobulin administration [11]. PCR can prove instrumental for monitoring response to immunoglobulin administration and predicting relapse.

## Conclusion

PVB19 presence in the blood and bone marrow should be investigated in neutropenia patients receiving disease-modifying antirheumatic drugs. New studies are required in order to further explore this association. In cases where agranulocytosis manifests from the 15^th^ day of RTX treatment, unlike typical LON timing, a hemogram must be conducted prior to reinitiating the infusion.
